# Influence of the Mechanical Properties of Third-Generation Artificial Turf Systems on Soccer Players’ Physiological and Physical Performance and Their Perceptions

**DOI:** 10.1371/journal.pone.0111368

**Published:** 2014-10-29

**Authors:** Javier Sánchez-Sánchez, Jorge García-Unanue, Pedro Jiménez-Reyes, Ana Gallardo, Pablo Burillo, José Luis Felipe, Leonor Gallardo

**Affiliations:** 1 School of Sport Sciences, UCAM, Universidad Católica San Antonio, Murcia, Spain; 2 IGOID Research Group, University of Castilla-La Mancha, Toledo, Spain; 3 Sport Sciences Institute, Camilo José Cela University, Villafranca del Castillo, Madrid, Spain; 4 School of Sport Sciences, European University, Villaviciosa de Odón, Madrid, Spain; Universidad Europea de Madrid, Spain

## Abstract

The aim of this research was to evaluate the influence of the mechanical properties of artificial turf systems on soccer players’ performance. A battery of perceptive physiological and physical tests were developed on four different structural systems of artificial turf (System 1: Compacted gravel sub-base without elastic layer; System 2: Compacted gravel sub-base with elastic layer; System 3: Asphalt sub-base without elastic layer; System 4: Asphalt sub-base with elastic layer). The sample was composed of 18 soccer players (22.44±1.72 years) who typically train and compete on artificial turf. The artificial turf system with less rotational traction (S3) showed higher total time in the Repeated Sprint Ability test in comparison to the systems with intermediate values (49.46±1.75 s *vs* 47.55±1.82 s (S1) and 47.85±1.59 s (S2); *p*<0.001). The performance in jumping tests (countermovement jump and squat jump) and ball kicking to goal decreased after the RSA test in all surfaces assessed (*p*<0.05), since the artificial turf system did not affect performance deterioration (*p*>0.05). The physiological load was similar in all four artificial turf systems. However, players felt more comfortable on the harder and more rigid system (S4; visual analogue scale = 70.83±14.28) than on the softer artificial turf system (S2; visual analogue scale = 54.24±19.63). The lineal regression analysis revealed a significant influence of the mechanical properties of the surface of 16.5%, 15.8% and 7.1% on the mean time of the sprint, the best sprint time and the maximum mean speed in the RSA test respectively. Results suggest a mechanical heterogeneity between the systems of artificial turf which generate differences in the physical performance and in the soccer players’ perceptions.

## Introduction

Soccer is a sport defined by periods of high intensity, with phases of recovery in which there are interspersed actions such as jumps, runs and kicks with frequent accelerations and direction changes [1]–[4]. The international impact of this sport has resulted in a high number of studies concerning the player’s internal factors which have an impact on their performance. Thus, physical parameters [5]–[7], physiological parameters [8], [9] and psychological parameters [10] have been demonstrated as being influential in the internal variables of soccer players’ performance. On the other hand, the effect of external variables such as nutritional supplements [11], recovery treatment [12], the temperature [13] or the situational variables are increasingly studied.

One of these external variables that has an influence on the game is the sports surface. It has been seen that the condition of the playing field is also a factor which affects the soccer player’s performance [14]. The surface–player interaction has been linked to the association between the different kinds of surfaces and sports footwear [15]–[18]. The first comparative studies between surfaces focused on the lower injury rates associated with natural grass compared to those associated with artificial turf [19], [20]. However, the artificial surface has undergone qualitative improvement and injury rates between the two surfaces have been levelled [21], [22]. Nonetheless, professional players still perceive there to be a higher risk of injury on artificial turf [23]. Despite this, a longer fibre, an improvement of the mechanical properties of the infill and the perfection of the structural component of support, have been enough to gain the backing of the Fédération Internationale de Football Association (FIFA) and the European Committee for Standardization (CEN) for these kind of surfaces to be used for playing soccer, through the corresponding quality control test adjusted to rigorous requirements [24]–[26].

The controversy provoked between the use of natural or artificial surfaces in soccer led to studies about their effects on the athlete’s performance [14], [27]–[32]. In sprint actions, these researches revealed similar times and speeds on both surfaces [14], [27], [29], [30], or even better on artificial turf [31]. Furthermore, isolated exercises on artificial turf do not induce higher fatigue or a delay in recovery [14], [32]. Nevertheless, despite this, some studies have shown a higher perceived physical effort by players when they play on artificial turf [27], [33], [34], as well as a higher level of lactate and heart rate in sub-maximum runs [28]. On the other hand, technical parameters in matches played on artificial turf evidenced a decrease in the incidences of slipping and an increase in the number of short passes [27]. The impact speed of the ball is another technical variable affected by the type of surface. [35]. Brito, Krustrup and Rebelo [36] extended the study framework to sand, asphalt and artificial turf surfaces. This work found a higher physical load in the sand compared to the artificial turf and the asphalt, due to a lower capacity to run at high speed on this surface [37]. These studies were undertaken due to the differences between the mechanical properties of each kind of surface and their effect on the performance. In fact, the increment of the traction coefficient between the footwear and the surface of up to 0.82 has demonstrated an improvement in the soccer player’s performance in lineal accelerations and circular sprints [38]. Similar researches show that a higher energy restitution associated with a higher rigidity contributes to an improvement in the economy of running [39], [40]. Lastly, McGhie and Etemma [41] highlight force reduction as an important factor in an athlete’s performance and security due to the reduction of the impact peaks during the sports practice.

As a result, the evolution that has taken place in artificial turf pitches has led to a high heterogeneity in the construction structures of third-generation artificial turf surfaces [42]. In fact, Potthast, Verhelst, Hughes, Stone and De Clercq [35] showed that the differences between various systems of artificial turf can be even higher than those between natural grass and artificial turf. Recent studies have demonstrated the influence of the structural component on the mechanical properties of an artificial turf soccer field, showing differences in levels of force reduction, standard vertical deformation and rotational traction according to the system installed [43], [44]. Despite this, no research has yet compared the effect of this mechanical variability in artificial turf pitches on the players’ performance and physiological responses. Therefore, the aim of this research is to establish the influence of the mechanical properties of artificial turf pitches with different structures on the physical performance, physiological responses and perceptions of amateur soccer players.

## Methods

### Subjects

Eighteen amateur soccer players from the same team (regional competitions) participated in the study (Mean: 22.44 SD: ±1.72 years; 73.74±8.47 kg; 175±6 cm; 14.74±4.15% of body fat). All of them have at least five years (6.28±2.13 years) previous experience as soccer players on artificial turf and they train two hours per day, three to four days per week, with one weekly competition, currently. None of the participants had any known cardiopulmonary disease or took any medicine during the study. Moreover all of them confirmed they had passed the medical examination required to play soccer.

At the beginning, the sample was composed of 20 players, but the two goalkeepers were rejected due to the difference in their movement with the outfield players. The rest of the players completed every test. All players were informed about the possible risk associated with this study, and they signed the informed consent form to be able to participate in the study. The Clinical Research Ethical Committee of Castilla-La Mancha Health Service (Spain) approved this study (n.13/10) basing on the last version of the Helsinki Declaration.

### Experimental design

A pilot test was conducted on an artificial turf pitch in order to guarantee that players became accustomed to the different tests included in the study protocol. Each player repeated the test on each of the four systems of third-generation artificial turf selected, following the sequence set up by the numeration of the surface (from System 1 to System 4). The four artificial turf pitches selected (located in Madrid and Castilla-La Mancha, which are central regions of Spain) had differences in their structural components of support (sub-base and elastic layer). Table 1 shows the characteristic of the artificial turf systems selected.

**Table 1:** Characteristics of the artificial turf selected.

Characteristics	System 1	System 2	System 3	System 4
Fibre				
Fibre material	Polyethylene	Polyethylene	Polyethylene	Polyethylene
Fibre type	Monofilament	Monofilament	Monofilament	Monofilament
Pile height	60 mm	45 mm	60 mm	45 mm
Dtex	12000	12000	12000	12000
Infill				
Sand material	Quartz	Quartz	Quartz	Quartz
Granulometry	0.3–0.8 mm	0.3–0.8 mm	0.3–0.8 mm	0.3–0.8 mm
Quantity	20 Kg/m^2^	15 Kg/m^2^	20 Kg/m^2^	15 Kg/m^2^
Rubber material	SBR	SBR	SBR	SBR
Granulometry	0.5–2.5 mm	0.5–2.5 mm	0.5–2.5 mm	0.5–2.5 mm
Quantity	13 Kg/m^2^	8 Kg/m^2^	13 Kg/m^2^	8 Kg/m^2^
Support structure				
Sub-base material	Compacted Gravel	Compacted Gravel	Asphalt	Asphalt
Elastic layer	No	Yes	No	Yes
Elastic layer thickness	-	23 mm	-	12 mm

**Figure 1:**
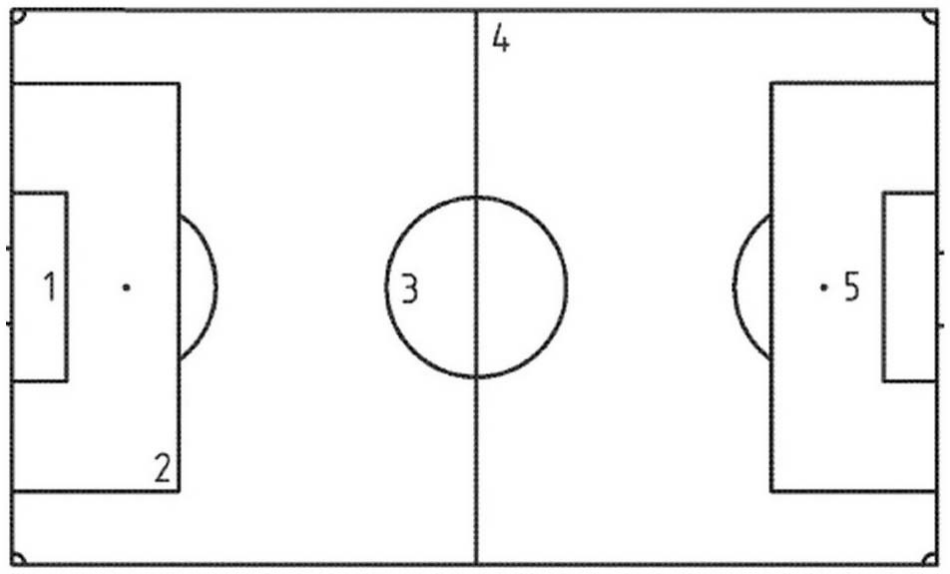
Test zones according to regulation EN 15330-1: 2014.

The mechanical properties related to the surface–player interaction were assessed in the four artificial turf systems selected for this study. *In situ* tests were conducted on the four surfaces which were followed by the protocols and specifications presented in norm EN 15330-1∶2014 and previous studies [43], [44]. The analyzed variables were force reduction (*FR-%*), standard vertical deformation (*StV-mm*), energy restitution (*ER-%*) and rotational traction *(RT-N·m*). The first three variables are related with the surfaces’ response to an impact, and they were evaluated using an Advanced Artificial Athlete (Deltec Metaal, Duiven, Holland), under the procedures stipulated by the norm EN 14808∶2005 and EN 14809∶2005. A mass with an incorporated spring, weighing in total 20 kg, was dropped on the surfaces. The acceleration of the mass from the output until after surface impact was transmitted through a data acquisition box (data acquisition device case) to a laptop with an informatics software (G-Force v.3.03, DeltecMetaal, Duiven, Holland) to extrapolate the collected data to the variables evaluated. This procedure was repeated two more times at intervals of 60±10 s, resulting in a total of three impacts. For the statistical analysis, the mean value between the second and the third impact was registered. In turn, this was done inside the five zones specified by the regulations EN 15330-1∶2014 (Figure 1) in two different testing positions separated by more than 100 mm.

The last variable informed about the resistance offered by the surface to perform a spin measured in Newton per meter (N·m). For this test, a Rotational Resistance Tester (Deltec Metaal, Duiven, Holland) was used, following the procedure indicated by the regulation EN 15301-1∶2014. The test zones were those indicated by the norms EN 15330-1∶2014 (Figure 1). A mass of 46 kg with a base of soccer cleats was dropped to the surface from a height of 60±5 mm. Once the base was anchored on the surface, a dynamometric key was turned 45° at a nominal rotation speed of 12 r/min. This procedure was repeated five times in each of the zones shown in Figure 1, with a distance of at least of 50 mm between each test point.

The geographical proximity of the four soccer pitches with artificial turf guaranteed similar climatic conditions in each of the trials. A period of 72 h was established between the different sessions in order to guarantee the total recovery of the players. Tests were carried out under climatic conditions of 18–22.5°C temperature and 20–35% humidity, during May.

### Experimental protocol

The day before the experimental test, it was recommended to the players to not carry out any kind of exhausting activity, as well as to maintain similar eating habits; they were also advised to use the same footwear in all four systems being assessed. Players arrived at the test soccer pitches at 09∶00 a.m. A Global Positioning System (GPS, Spi Pro X, GPSports, Australia) was incorporated on each soccer player’s back. Before the beginning of the different tests, participants carried out a standard warm-up which had exercises such as 5 min of continuous run, 5 min of exercises of articulation mobility and three sprints of 30 m, increasing the intensity, with a recovery process of 2 min between each test. Stretching exercises were not carried out during the warm-up. At 10∶00 a.m. players started the different performance tests, while they were verbally instructed to apply the maximum effort during the tests.

### Repeated sprint ability test (RSA)

Players performed three maximum sprints of 30 m with intermediate measurement points at 5 and 10 m before carrying out the RSA test. The best time achieved in these sprints was selected for the analysis.

Players carried out the RSA test after 15 min of recovery. The test consisted of six 40 m sprints (20+20 m) [45]. Participants sprinted 20 m, then they turned on one line and then ran back to the starting line at maximum intensity. The deceleration took place one metre after passing the starting line. After 20 s of active recovery, players repeated the same procedure. There had to be a gap of 5 seconds before the start of the next sprint, and the researcher carried out the countdown. A system of four pairs of photocells (Microgate, Bolzano, Italy) was placed along the sprint zone, which collected time at 5, 10, 20, 30 and 40 m with a sensibility of 0.001 s. If the time of the first sprint of the RSA test was higher (increment higher than 5%) than the best individual sprint done before the beginning of the test, it was not considered valid [5], [46], [47], therefore players had to repeat the test after 5 min of recovery. The best time of sprint (RSA_BEST_), the mean time (RSA_MEAN_), the total time (RSA_TT_), the percentage of decreasing (%Dec) and the difference between the best and worst sprint during the RSA test (%Diff) were calculated [45], [46], [48]. The %Dec ((mean time/best time×100) –100) has been identified as the most valid and reliable method for assessing fatigue in these kinds of tests [12], [48], while recent studies have demonstrated a high correlation with the %Diff [46]. The maximum peak of speed (V_MAX_), the maximum mean speed of run (V_MEAN_) and the heart rate of players during the RSA were monitored by GPS of 10 HZ, which has been demonstrated as being a valid and reliable tool in the collection of these variables [1], [49].

A minute after finishing the RSA test, a specialized researcher took a sample of 5-µL of blood lactate from the fingertip of the participants using a portable device (Lactate Scout, SensLab GmbH, Leipzig, Germany). This procedure was repeated 3 minutes after finishing the RSA test.

### Vertical jumping

Countermovement jumps (CMJ) and squat jumps (SJ) were done before and after the RSA test, using an infrared system (Optojump Next, Microgate, Bolzano, Italy). Participants had to keep their hands on their hips to eliminate the influence of arm movement on jump performance. Every player did two jumps in each modality before (with 2 min of recovery between jumps) and after the RSA test. The best of them was selected for the statistical analysis.

In the same way, players carried out the fifteen seconds maximal jump test to assess the performance deterioration in their jumping action. For this test, participants had to stand upright with feet apart at shoulder width and with their hands on their hips in the same way as in the CMJ. At the start signal, subjects flexed their knees and carried out a maximum performance of jumps for 15 s, landing with both feet at the same time. The maximum height in cm, % Dec (100–(mean jump/best jump×100)) [12] and the output power [(62.5×50.3×jump height+body mass)–2184.7]×number of jumps] [50] were calculated for this test.

### Kicking from goal

Players had to kick, at the fastest speed possible, a stationary ball placed on the penalty point at 11 m from the goal. The ball used for this test had the FIFA-approved certification and a level of pressure according to the standards established by the organisation. Players kicked twice before and after the RSA test, with a rest of 1 min between each kick. The fastest kick in each round was selected for the statistic analysis. The ball speed (Km/h) was collected by means of a Stalker ATS System TM radar (Radar Sales, MN, US) placed behind the goal and pointing at the ball.

### Visual analogue scale

The perceived effort and the fatigue experience on each artificial turf system were evaluated by means of a visual analogue scale (VAS) questionnaire used in previous studies [27], [36]. The questionnaire included six questions and it was applied once the session on each artificial turf system was finished. Players answered on a horizontal line of 100 mm where 0 was “nothing, hard/tired/comfortable” and 100 was “very hard/tired/comfortable.” The questions were: “How can you classify the effort made during this session?” (VAS1); “How tired are you at this moment?” (VAS2); “How difficult have you found it to do a turn or change direction?” (VAS3); “How did you feel during the jumps?” (VAS4); “How did you feel during the run?” (VAS5); “In general, how have you felt during the session?” (VAS6).

### Statistical analysis

Results are presented as mean and standard deviation (SD). The verification of the normality and homogeneity of the variances was assumed by means of the Kolmogorov–Smirnov test and the Leven’s statistic. The influence of artificial turf systems on the selected variables was assessed through a variance analysis (ANOVA) and the differences between means were identified using a post hoc Bonferroni procedure. The comparison between results collected in the vertical jumping test and kicking ball tests before and after the RSA test were developed through a two-way ANOVA test (artificial turf system×time). A lineal regression analysis was used for the mechanical properties of the artificial turf systems as independent variables and the results from the different performance test as dependent variables. The *StV* was omitted due to its high correlation with *FR* (*r = *0.99*; p*<0.0001*)*, deleting possible problems of multicollinearity. Data were analyzed with the statistic software SPSS v 20.0. The level of significance was established at *p*<0.05.

## Results

### Mechanical properties of the surface

Table 2 shows the mechanical differences between the four selected systems of artificial turf. System 2 (composed of a compacted gravel sub-base and elastic layer) presented the highest value of *FR* (69.83±1.18%) and *StV* (6.56±0.37 mm) in comparison with the rest of the systems (*F* = 451.63 y *F* = 326.92; *p*<0.001). The highest results of *ER* and *RT* were identified in System 4 (50.50±2.19% y 54.60±4 N·m, respectively) which incorporated an asphalt sub-base and elastic layer.

**Table 2:** Mechanical properties of the selected artificial turf systems.

	System 1 (a)		System 2 (b)		System 3 (c)		System 4 (d)		*F*	*p*
*FR* (%)	51.30	(2.52)^b,c^	69.83	(1.18)	60.10	(2.04)^b^	48.07	(3.67)^a,b,c^	451.63	<0.001
*StV* (mm)	3.63	(0.43)^b,c^	6.56	(0.37)	4.68	(0.45)^b^	3.43	(0.48)^b,c^	326.92	<0.001
*ER* (%)	42.63	(1.50)^c,d^	42.07	(1.46)^c,d^	43.90	(1.45)^d^	50.50	(2.19)	161.26	<0.001
*RT* (N·m)	45.56	(2.84)^b,c,d^	42.44	(3.14)^a,d^	41.72	(2.81)^a,d^	54.60	(4)	83.81	<0.001

System 1: Compacted gravel sub-base without elastic layer; System 2: Compacted gravel sub-base with elastic layer; System 3: Asphalt sub-base without elastic layer; System 4: Asphalt sub-base with elastic layer.

*FR = *Force Reduction; *StV = *Standard Vertical Deformation; *ER = *Energy Restitution; *RT = *Rotational Traction.

a,b,c,d Significant differences with the system indicated (*p*<0.05).

### Repeated sprint ability test

The global performance analysis done in the RSA test (Table 3) on the four artificial turf systems revealed a significantly lower RSA_MEAN_ of sprint and RSA_TT_ (*F* = 4.214 and *F* = 4.216; *p*<0.01) in Systems 1 and 2 in comparison with System 3, which presented the lowest level of *RT*. On the other hand, the time of the best sprint was 7.38±0.35 s on System 1 and 7.74±0.29 s on System 3 (*F* = 4.002; *p<0.05*). Speed parameters of run and heart rate during the RSA test were not affected by the system of artificial turf. Also the performance deterioration in the RSA test was similar in all four artificial turf systems.

**Table 3:** Time speed and heart rate values in the RSA test in the four artificial turf systems.

	System 1 (a)		System 2 (b)		System 3 (c)		System 4 (d)		*F*	*p*
Time										
RSA_MEAN_ (s)	7.93	(0.30)^c^	7.97	(0.26)^c^	8.24	(0.29)	8.02	(0.25)	4.214	0.009
RSA_BEST_ (s)	7.38	(0.35)^c^	7.50	(0.26)	7.74	(0.29)	7.51	(0.32)	4.002	0.011
RSA_TT_ (s)	47.55	(1.82)^c^	47.85	(1.59)^c^	49.46	(1.75)	48.14	(1.48)	4.216	0.009
% sprint Dec 40 m	7.44	(1.74)	6.40	(2.45)	6.53	(2.10)	6.90	(2.85)	0.681	0.567
% sprint Diff 40 m	13.42	(2.99)	12.20	(4.63)	11.37	(3.87)	12.08	(4.03)	0.782	0.508
Speed										
V_MAX_ (km/h)	26.84	(1.06)	27.13	(1.66)	26.69	(2.28)	26.72	(1.22)	0.267	0.849
V_MEAN_ (km/h)	24.78	(0.71)	24.67	(1.06)	24.19	(0.72)	24.44	(0.78)	1.635	0.190
% speed Dec	7.62	(2.55)	8.94	(3.29)	9.02	(4.62)	8.45	(2.24)	0.649	0.586
% speed Diff	14.52	(3.48)	15.99	(4.80)	15.71	(5.36)	15.39	(4.14)	0.342	0.795
Heart rate										
Peak HR (b.p.m)	184.00	(12.90)	185.18	(12.48)	183.13	(13.19)	184.78	(11.72)	0.083	0.969

System 1: Compacted gravel sub-base without elastic layer; System 2: Compacted gravel sub-base with elastic layer; System 3: Asphalt sub-base without elastic layer; System 4: Asphalt sub-base with elastic layer.

RSA* = *Repeated Sprint Ability.

a,b,c,d Significant differences with the indicated system (*p*<0.05). Data are presented as mean (SD) in 18 soccer players.

**Figure 2:**
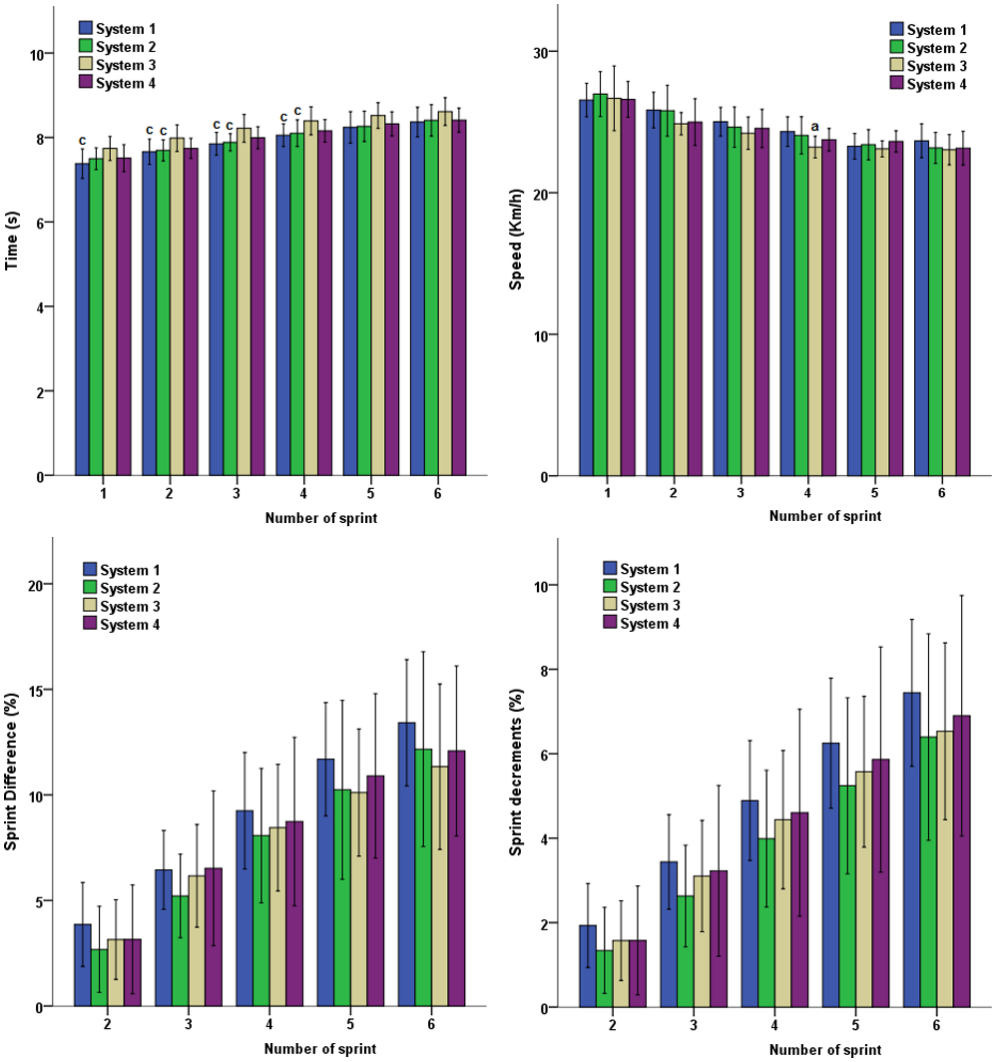
Time, speed and performance deterioration profile in the sprints (%Diff and %Dec) of the RSA test (6×40 m) on different artificial turf systems.

In the analytic comparison per number of sprints, the times achieved in the four first sprints of the RSA test were significantly higher on the system with an asphalt sub-base and elastic layer (System 3) than on Systems 1 and 2 (Figure 2: *p*<0.05). Therefore, the maximum speed achieved in the fourth sprint was 23.23±0.78 km/h on System 3 and 24.32±1.05 km/h on System 1 (*F* = 3.552; *p*<0.05). The absence of significant differences in the variables of %Diff and %Dec indicates that the game surface has no additional influence on the development of fatigue during the RSA test (*p*<0.05).

**Figure 3:**
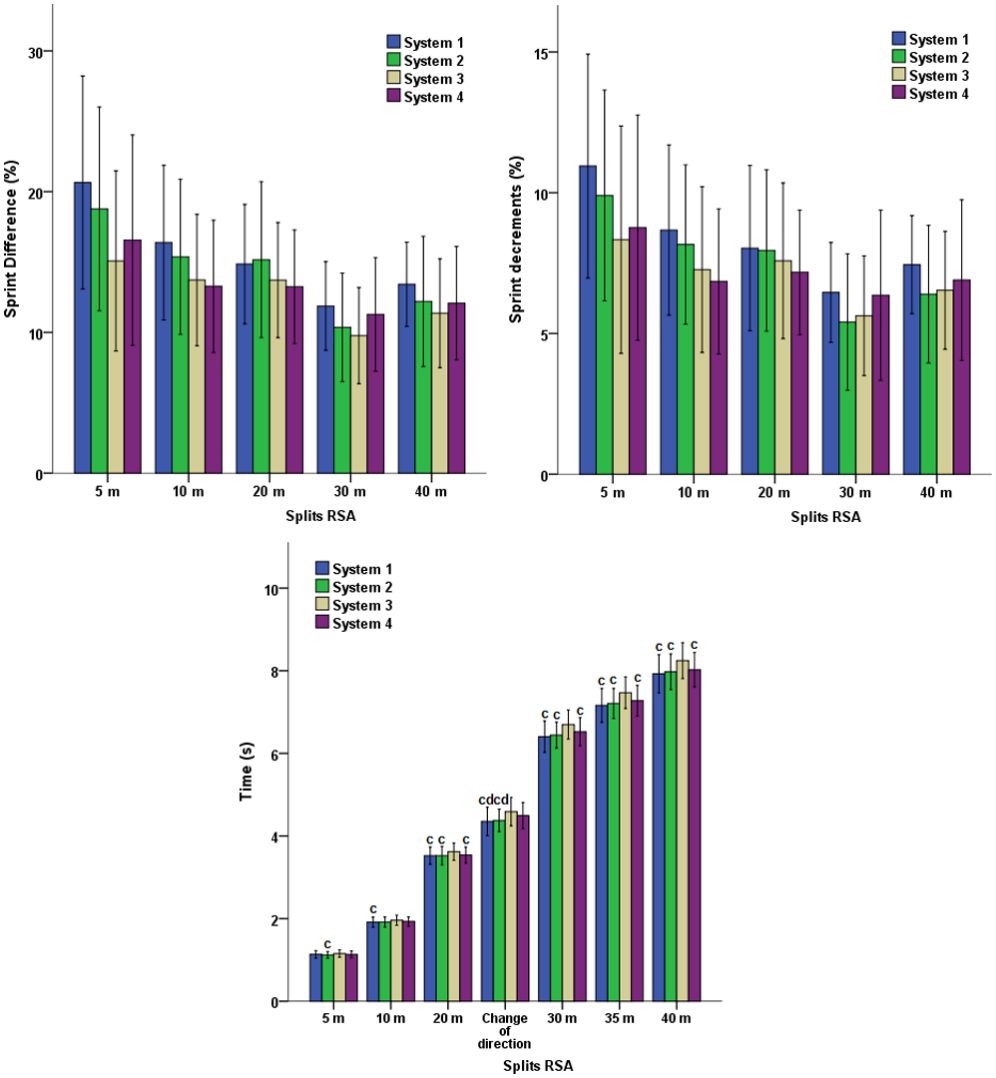
Performance and deterioration of times and percentage (%Dec and %Diff) in sprints of the RSA test specified by distance splits on different artificial turf systems.

The split-distance RSA test revealed superior times on System 3 compared to the other artificial turf systems. These differences were more significant from the 20 m (*p*<0.001; Figure 3). The direction changes presented significantly lower times in Systems 1 and 2, which evidenced intermediate values of *RT*, than Systems 3 and 4 with traction values in the lower and upper end of the sample, respectively (4.35±0.34 s y 4.38±0.27 s *vs* 4.59±0.34 s y 4.50±0.32 s; *F* = 11.553; *p*<0.001). Once again, the fatigue index (%Diff and %Dec) did not significantly differ between the evaluated systems of artificial turf (*p>*0.05), negating the influence of the game surface on this variable.

Lactate values in blood after the RSA test were not affected by the game surface. The collected samples at 1 minute (S1: 12.92±2.27 mmol·L^−1^; S2: 12.44±2.42 mmol·L^−1^; S3: 11.04±2.31 mmol·L^−1^; S4: 11.80±2.53 mmol·L^−1^) and 3 min after the ending of the RSA (S1: 12.98±2.51 mmol·L^−1^; S2: 12.97±2.98 mmol·L^−1^; S3: 11±1.86 mmol·L^−1^; S4: 11.06±2.46 mmol·L^−1^) test were similar for the four analyzed systems (*p*>0.05).

### Vertical jumping

The system of artificial turf did not generate any influence upon either the jumping height or the power generated in the CMJ and SJ test and the 15 s test (*p*>0.05; Table 4). After the RSA test a significant deterioration in the jumping height and the power generated in the CMJ and SJ was detected in all the evaluated artificial turf systems (*p*<0.05). The interaction between the artificial turf system and the performance deterioration reveals significant differences in the fatigue index (%Diff) in the ANOVA test of the CMJ (F = 2.942; *p*<0.05) and the SJ test (F = 3.539; *p*<0.05). Results showed a higher fatigue index in the systems with higher levels of *FR* and *StV*. However, the post hoc analysis did not confirm the significant differences between pairs (*p>*0.05). On the other hand, in the 15 s jumping test, players confirmed a significantly greater deterioration in the height of the jump on the system with the biggest *ER* (System 4; *F* = 3.188; *p*<0.05). Finally, the variation percentage of the applied power (%Diff Power output) in the CMJ and SJ test after the RSA test was not affected by the type of artificial turf system (*p>*0.05).

**Table 4:** Valuation of the jumping tests CMJ, SJ and 15 s on the different artificial turf systems.

	System 1 (a)		System 2 (b)		System 3 (c)		System 4 (d)		*F*	*p*
CMJ										
CMJ_PRE_ height (cm)	36.53	(4.40)*	35.46	(5.38)*	36.36	(5.72)*	36.92	(5.11)*	0.249	0.862
CMJ_POST_ height (cm)	28.31	(4.46)	26.03	(4.87)	26.53	(5.21)	30.09	(5.85)	2.248	0.091
% Diff CMJ height	22.49	(7.45)	26.12	(10.67)	27.05	(7.62)	18.47	(11.26)	2.942	0.040
Power output_PRE_ (W)	3809.35	(515.98)*	3697.76	(491.98)*	3852.28	(553.17)*	3832.01	(528.95)*	0.294	0.830
Power output_POST_ (W)	3295.37	(527.67)	3108.42	(492.59)	3237.83	(547.07)	3404.92	(577.83)	0.919	0.437
% Diff Power output	13.66	(5.13)	15.91	(7.14)	16.09	(5.24)	11.25	(7.10)	2.286	0.087
SJ										
SJ_PRE_ height (cm)	28.49	(4.39)*	29.69	(4.03)*	29.30	(5.46)*	29.60	(4.55)*	0.238	0.869
SJ_POST_ height (cm)	24.57	(4.28)	23.22	(4.35)	23.14	(4.69)	25.09	(3.53)	0.922	0.436
% Diff SJ height	13.74	(7.50)	21.71	(9.86)	20.72	(9.85)	14.66	(8.50)	3.539	0.019
15 s Test										
Mean jump height (cm)	28.68	(3.66)	28.25	(4.51)	28.55	(4.92)	29.34	(3.65)	0.211	0.888
Número de saltos (n)	17.18	(2.13)	15.53	(1.33)	16.56	(2.10)	15.94	(1.80)	2.554	0.063
Power output (W)	57030.07	(11577.78)	50598.42	(9234.74)	55574.10	(10710.92)	53522.08	(9962.71)	1.218	0.310
% jump Dec	10.58	(2.84)	10.37	(2.86)^ d^	11.57	(3.93)	13.66	(4.26)	3.188	0.030

System 1: Compacted gravel sub-base without elastic layer; System 2: Compacted gravel sub-base with elastic layer; System 3: Asphalt sub-base without elastic layer; System 4: Asphalt sub-base with elastic layer.

CMJ = Counter Movement Jump; SJ* = *Squat Jump; 15 s Test* = *Fifteen seconds maximal jump test.

Data are presented as mean (SD) in 18 soccer players.

*Significant differences between pre- and post-RSA (*p*<0.05).

a,b,c,d Significant differences with the indicated system (*p*<0.05).

### Goal kicking

The RSA test generated a significant deterioration of the kicking speed of the ball in each of the four systems of artificial turf (S1: 7.34±5.18%; S2: 11.62±7.67%; S3: 11.79±6%; S4: 7.55±6; *p*<0.05). However, there was no significant interaction between the game surface and the kicking speed of the soccer players (*p>*0.05), which showed a mean between the four systems of 102.54±8.87 km/h before the RSA test and 92.85±8.96 at the end of the test.

### VAS

**Figure 4:**
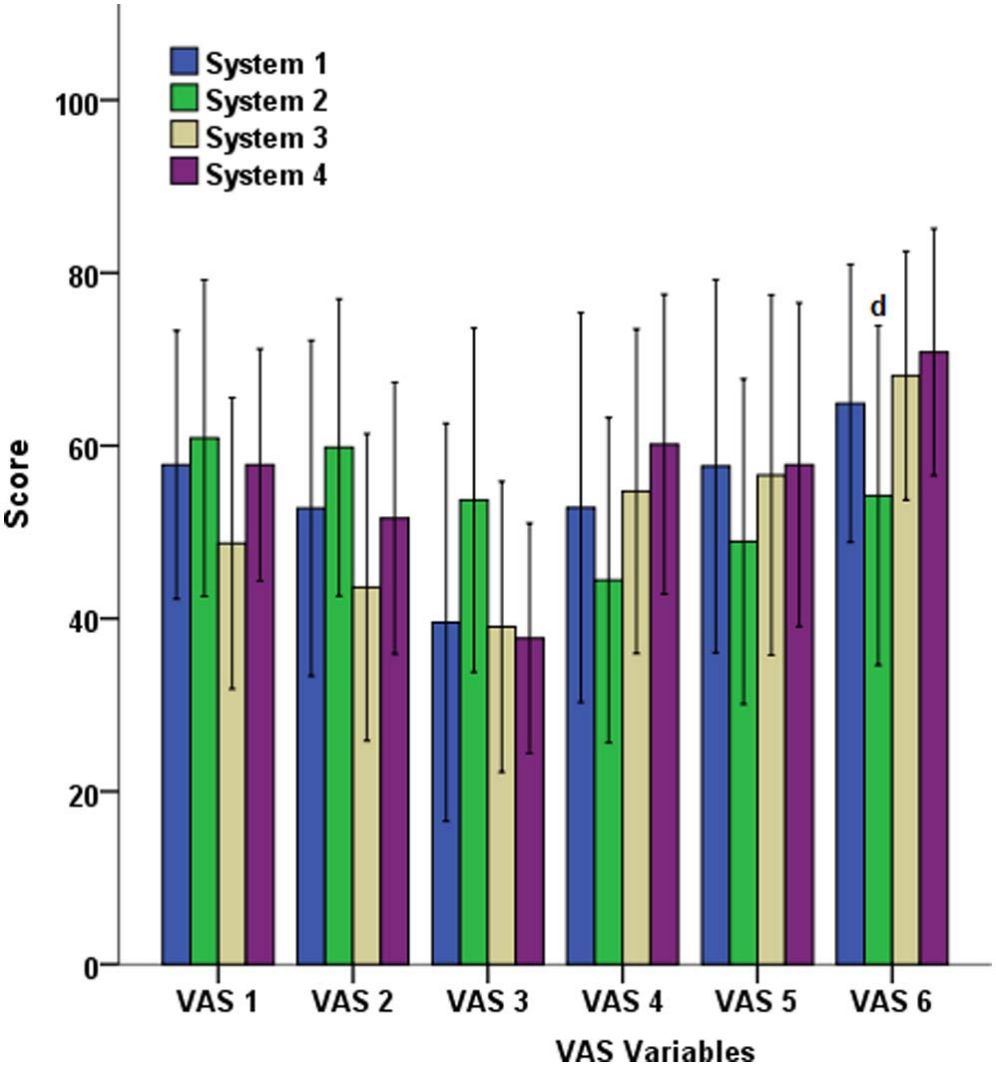
Results of the visual analogue scale (VAS) after the session on the artificial turf systems.

Once the session was ended, the perception of players regarding the effort parameters (VAS1), fatigue (VAS2), difficulty in direction changes (VAS3), jumps (VAS4) and run (VAS5) showed no differences between the systems of artificial turf (*p>*0.05; Figure 4). In contrast, at a general level, players reported a higher sense of comfort during the session developed on the system with fewer *FR* and *StV*, as well as with the highest levels of *ER* and *RT* (System 4), compared to System 2, which presented the biggest *FR* and *StV* and the lowest *ER* of the four systems (VAS6: 70.83±14.28 *vs* 54.24±19.63, respectively; *F* = 3.413; *p*<0.05).

The lineal regression analysis evidenced a significant influence of the mechanical properties of the systems of artificial turf on the performance in the RSA test (*p*<0.05; Table 5). In particular, the *ER* presented a positive influence on the RSA_MEAN_, RSA_TT_ y RSA_BEST_ (*p*<0.001) and a negative one on the maximum mean speed in this test (*p<*0.05). Moreover, the *RT* showed a negative influence on the RSA times (*p<*0.01). Therefore, this highlights the fact that the evaluated mechanical properties (*FR, ER and RT)* had a significant influence of 16.5%, 15.8% y 16.5% on RSA_MEAN_, RSA_BEST_ and RSA_TT_, respectively. Lastly, a higher *FR* was associated by players with a lower perceived comfort on the respective different artificial turf systems (*p*<0.05).

**Table 5:** Lineal regression analysis of the effect of the mechanical properties of the artificial turf system on the jump, sprint and perception parameters.

	CMJ(cm)	FatigueCMJ	SJ (cm)	FatigueSJ	Power output15 s (W)	% JumpDec 15 s	RSA_MEAN_ (s)	RSA_BEST_ (s)	RSA_TT_ (s)	% SprintDec	V_MAX_RSA (km/h)	V_MEAN_RSA (Km/h)	% SpeedDec	VAS6
*FR* (%)	–0.075	–0.025	0.071	0.277	–513.887	–0.029	–0.013	–0.009	–0.079	–0.044	0.032	0.020	0.042	–1.001*
	(0.142)	(0.260)	(0.127)	(0.246)	(285.729)	(0.097)	(0.008)	(0.008)	(0.046)	(0.064)	(0.044)	(0.023)	(0.090)	(0.455)
*ER* (%)	0.136	0.843	0.160	1.299	321.772	0.570	0.114***	0.120**	0.684***	–0.191	–0.110	–0.217*	0.341	3.247
	(0.611)	(1.120)	(0.547)	(1.060)	(1229.840)	(0.418)	(0.033)	(0.036)	(0.196)	(0.276)	(0.190)	(0.098)	(0.386)	(1.997)
*RT* (N·m)	–0.102	–1.189	0.008	–0.930	–851.791	–0.167	–0.093**	–0.093**	–0.558**	0.091	0.094	0.159	–0.191	–2.528
	(0.510)	(0.935)	(0.456)	(0.885)	(1027.172)	(0.349)	(0.027)	(0.030)	(0.164)	(0.230)	(0.159)	(0.082)	(0.323)	(1.653)
Constant	39.199*	42.006	17.627	–13.480	108482.935**	–4.629	7.981***	6.992***	47.882***	13.704	25.660***	25.742***	–0.397	93.033
	(16.119)	(29.543)	(14.417)	(27.956)	(32440.648)	(11.028)	(0.864)	(0.952)	(5.183)	(7.278)	(5.019)	(2.598)	(10.190)	(52.703)
R^2^	0.012	0.121	0.011	0.142	0.054	0.130	0.165	0.158	0.165	0.031	0.012	0.071	0.030	0.144

CMJ = Counter Movement Jump; SJ* = *Squat Jump; 15 s* = *Fifteen seconds maximal jump test; RSA* = *Repeated Sprint Ability; VAS* = *Visual Analogue Scale.

*FR = *Force Reduction; *ER = *Energy Restitution; *RT = *Rotational Traction.

Significant influence *p<0.05 **p<0.01 ***p<0.001.

## Discussion and Conclusions

This research exposes the influence of the mechanical heterogeneity of the artificial turf system on the sport performance, which is evaluated through specific tests. With regards to physical tests, this study included a combination of performance measures and physiological responses similar to recent researches that compared artificial turf with natural grass [14], [32]. However, this is the first research which applies these measures on several different systems of artificial turf. Previous studies defend a lack of connection between the mechanical devices and the mechanical properties perceived by the players, due to the lack of the human movement auto-regulation [51], [52]. The surface with a compacted gravel sub-base and elastic layer had the highest levels of *FR* and *StV*, coinciding with previous studies which assessed the influence of the structural components of support on the mechanical properties of the surface [44], in spite of this; this system met the regulation requirements. The inclusion of the *ER* and *RT* provides more details about hardness, the capacity of absorption and turn resistance from the surface, respectively. This property presents an inverse relationship with the impact reduction (*r* = –0.665; *p*<0.01) and this differs depending on the system of artificial turf introduced, due to the heterogeneity and the state of conservation of the structural components used [52]–[55].

The results of the current research show differences between the systems of artificial turf in the performance of the RSA test. The mean, total and best times identified as the main indicators of the performance in this test [56]–[58], show significant differences based on the surface assessed. The systems with intermediate values adapted to the normative requirement specified for *RT*, presented faster sprint times (mean, total and best) than the system with a lower index of *RT* and System 4, which is over the limit of 50 N·m according to the regulations. Similarly, Luo and Stefanyshyn [38] concluded that increases in the traction levels up to a certain threshold came from systematic advantages in the performance of sprints and accelerations, but these improvements were not detected in the performance from a certain threshold as it is observed with System 4 in this work. Moreover, higher levels of *RT* constitute a risk injury factor in the lower limbs of the players [59]. The inclusion of a direction change of 180° and the split analysis in the RSA test demonstrated that optimum levels of *RT* improve the lineal performance in the sprints, and also facilitate the capacity to change direction, because the differences between the artificial turf systems became more evident from the turns of 180°. Previous researches explained this improvement as being a result of less slipping by players on a surface with adequate rotational traction [29], [60], [61]. Gains et al. [30] found similar sprint speeds between artificial turf and natural grass, but found direction changes to be faster on artificial surfaces. Data from this work show the same tendency in third-generation systems of artificial turf in the times collected for the direction change in the RSA test, which presented significant differences between them, while changes in the speed were not observed. However, the lineal regression analysis revealed a negative influence of the *ER* of the surface on the V_MEAN_. In addition, the sum of the mechanical properties analyzed in the current study has demonstrated a effect of the 7.1% on the V_MEAN_ achieved by players during the RSA test. This analysis allows quantifying the limited influence of the mechanical properties of the surface on the maximum speed, discovered in previous studies that compared artificial turf with natural grass [14]. McGhie and Etemma [41] evidenced an influence of different types of artificial turf on the impact forces and the time of contact on soccer players in two run tests, but they did not take into account the traction properties of the surface. The comparison with these kinds of studies shows that the differences between natural grass and artificial turf are similar to the differences found between the different systems of third-generation artificial turf.

The lineal regression also shows the influence of *ER* on the performance in the RSA test. The analysis reveals that if the rigidity is excessive, it provokes an increase in the times of this test. However, the *FR* did not constitute a determinant variable on the performance in the repetitive sprints. This suggests that the percentages of *FR* were not high enough to generate an increase in the times derived from the reduction of the reaction forces as a result of the partial absorption of the energy applied [62]. The lack of differences in the performance, fatigue and physiological response in the RSA test between the systems of artificial turf with higher and lower impact-damping capacities, negates the hypothesis that softer surfaces require a greater exertion of energy [63], [64]. This is probably due to the limited magnitude of the differences in the force reduction between systems. The studies which explain a lower muscle-sinew efficiency [65] or a higher hip and knee flexion [66] as being indicative of a higher energy outlay on surfaces with a higher impact reduction, were performed on a sand surface with a much higher level of impact reduction than that detected on artificial turf. Current studies regarding artificial and natural grass present mixed results. Hughes et al. [14] did not detect any differences in the lactate levels in blood and heart rate after a simulated match. However, Di Michele et al. [28] evidenced differences in these parameters in a run test. In our case, the lactate samples in blood were collected after the RSA test, therefore results have to be compared cautiously, because several authors have demonstrated that lactate concentration is influenced by the activity done immediately before to the sampling [67]. The physiological load, which is similar between the systems of artificial turf, contrasts with the players’ perceptions on ending the session. In terms of general perceived effort, players reported higher comfort on the system with lower levels of *FR* with respect to the system with the biggest capacity for cushioning. Moreover, the regression demonstrated that the *FR* was the only variable which significantly influenced the users’ perception. The negative influence observed suggests that the softer surfaces seemed less comfortable for players during the different actions performed on the surface, in spite of the fact that there are no differences between the systems in the physiological answers from participants. This discovery coincides with Brito et al. [36] who show that the perceived demand is more related to the difficulty in running (external load) than to the physiological responses (internal load), even though the activity performed prior to the evaluation of the players’ perception was different in both studies. Similarly, Nédélec et al. [32] turn to the protocol used and the familiarisation with artificial turf in order to justify the lack of differences in the players’ perception of natural grass and artificial turf which was found in other studies [27]. However, in the current study every player was familiar with this surface and they showed different levels of perceived comfort regarding the artificial turf system. Regardless of the perceived effort, the mechanical properties evaluated showed an influence of 16.5%, 15.8% and 16.5%, on the RSA_MEAN_, RSA_BEST_ and RSA_TT_ respectively. These results highlight the importance of including the *ER* as a measurable parameter of the functionality of artificial turf soccer pitches, as well as the requirement of assessing the mechanical properties of the different surfaces in comparative studies.

The kicking ball to goal speed did not present differences in the four analysed systems. In all of them, players showed a significant deterioration of kicking speed after the RSA test. Nevertheless, the lack of interaction between the surface and the kick moment indicates that the artificial turf system has no additional influence on fatigue development. On the contrary, Potthast [68] found differences between two systems of artificial turf in the speed, accuracy and biomechanics parameters of kicking a ball. The utilization of two different systems of infill (rubber, or rubber and sand) in this research [68] may be the main reason for this discrepancy, because in the current study, the type of superficial structural components (fibre and infill) was identical in all four systems. Despite this, many more studies with biomechanical parameters are required to establish the determinant factors in ball kicking.

Finally, the different jumping tests (CMJ and SJ) performed before and after the RSA test evidence a significant deterioration in performance on the four systems with no interaction between the system of surface and the jump moment. This suggests a lower jumping capacity after a succession of sprints, regardless of the third-generation artificial turf system of employed to perform. This conclusion is similar to the results attained in other studies after applying a simulated protocol of soccer on natural grass and artificial turf [14]. The jumping test of 15 s evidenced a higher deterioration of performance (%Dec) on the system with harder and more rigid artificial turf. This system presented mechanical properties which were a long way from meeting the regulation requirements; therefore these results highlight the importance of meeting the regulations in order to guarantee optimum parameters of sport functionality. On the other hand, there are not any systems which exceed the regulation specifications established for the properties of the cushioning of surfaces. This suggests that the disadvantages offered by a soft surface [36], [63], [64], [66] are not apparent until a particular limit, in a similar way to the *RT*, although more studies on the influence of the surface on the repetitive jumping capacity are required to accept this hypothesis.

The variety of perceptual, physiological and physical tests included in the current study has permitted a comparison of the influence of different artificial turf systems and their mechanical properties on the players’ performance. Tests have focused on the main actions performed during a soccer match, such as repetitive sequences of explosive movements, sprints with direction changes and maximum jumps [12], [47]. However, in order to confirm whether the results collected are translatable to a real match these variables would need to be checked during a real-life game. Future studies regarding artificial turf systems should include more variability of artificial turf systems based on the superficial components as well as the different rates of mechanical properties so as to know the exact point in which the deterioration of performance begins and the risk of injury increases.

One of the possible limitations of this research was the implementation of an only data collection for each one of the artificial turf systems. The repetition of the test battery (with the same sample of players) in more selected fields with these characteristics will increase the consistency of the obtained results.

In conclusion, the different systems of artificial turf selected, based on their structural components of support, evidenced a mechanical heterogeneity which had influence on the physical parameters implicated in soccer performance. This mechanical variability placed artificial turf systems at different levels of compliance with specified regulatory requirements for artificial turf soccer pitches. The physical performance in sprint and jumps actions was influenced by the parameters of traction, stiffness and force reduction of the artificial turf systems. However, the physiological load remains invariable between the different systems, in spite of players’ perceived less comfort on the softer surfaces. The differences between artificial turf systems are similar to the variability between natural grass and artificial turf, so that control of the mechanical properties of the game surface is essential to ensure adequate sport functionality. The coaches must take into account that the practice of football on different structures of artificial turf does not modify the physiological parameters of the players, although differences are obtained in physical performance. This research provides the bases to ensure favourable conditions of the game that encourage sports participation at the community level. Future studies should incorporate technical and biomechanical parameters of different systems to complement the findings of this study.
